# Cabozantinib Cutaneous Toxicity—Comprehensive Review

**DOI:** 10.3390/life15010072

**Published:** 2025-01-09

**Authors:** Cristina Violeta Tutunaru, Dragos Ovidiu Alexandru, Sanda Amelia Dracea, Loredana Ungureanu, Liliana Gabriela Popa, Cristina Beiu

**Affiliations:** 1 Department of Dermatology, University of Medicine and Pharmacy of Craiova, 200349 Craiova, Romania; cristina.tutunaru@umfcv.ro; 2 Department of Dermatology, Clinical Emergency County Hospital of Craiova, 200642 Craiova, Romania; 3 Department of Medical Informatics and Biostatistics, University of Medicine and Pharmacy of Craiova, 200349 Craiova, Romania; 4 Department of Biophysics, University of Medicine and Pharmacy of Craiova, 200349 Craiova, Romania; 5 Department of Radiotherapy, Clinical Emergency County Hospital of Craiova, 200642 Craiova, Romania; 6 Department of Dermatology, “Iuliu Hațieganu” University of Medicine and Pharmacy, 400006 Cluj-Napoca, Romania; 7 Department of Oncologic Dermatology, “Elias” Emergency University Hospital, “Carol Davila” University of Medicine and Pharmacy, 020021 Bucharest, Romania

**Keywords:** cabozantinib, cutaneous toxicity, renal cell carcinoma, medication, adverse effects

## Abstract

Background: In the context of modern cancer therapy, the management of adverse effects of systemic therapies can lead to the avoidance of underdosing and withdrawal and increases in the quality of the therapeutic act and the quality of life. This review offers an overview of the skin-related toxicities associated with Cabozantinib, a multikinase inhibitor (MKI) that is approved for treating advanced kidney cancer, hepatocellular carcinoma, and medullary thyroid cancer. It covers the most common dermatological side effects, such as palmar–plantar erythrodysesthesia, stomatitis, hair alterations, xerosis, scrotal erythema, and subungual splinter hemorrhages. Additionally, this review includes suggested preventive strategies and management approaches based on the severity of these adverse effects.

## 1. Introduction

Tyrosine kinases (TKs) are a group of proteins that play a key role in the development of cancer by disrupting vital cellular functions, such as the cell cycle, growth, differentiation, apoptosis, and cell survival. Their action primarily involves the inhibition of the catalytic subunit of TKs. Cabozantinib functions as an ATP-competitive inhibitor targeting a variety of kinase receptors, including VEGF receptors (VEGFR 1, 2, 3), mesenchymal–epithelial transition (MET) receptor, hepatocyte growth factor receptor, and Axl (anexelekto receptor tyrosine kinase, linked to the GASP6 gene). By blocking these essential signaling pathways, Cabozantinib disrupts tumor blood vessel formation, angiogenesis, and the proliferation and spread of cancer cells. [1] Additionally, Cabozantinib targets other molecules such as ROS1, TRKA, TRKB, TYRO3 (FMS-like tyrosine kinase 3), the MER (TAM) family of receptor kinases, KIT, and FLT-3, which may contribute to its immune-modulating and anti-cancer effects [2,3,4].

Cabozantinib is a multikinase inhibitor (MKI) with a molecular weight of approximately 501.63 Da, and it has received approval for the treatment of advanced renal cell carcinoma (RCC) by the US FDA in 2016, hepatocellular carcinoma (HCC), and medullary thyroid cancer (FDA approval in 2012). Clinical trials have explored its efficacy in various cancers, including prostate cancer, non-small cell lung cancer, breast cancer, multiple myeloma, pancreatic cancer, salivary gland cancer, and Merkel cell carcinoma [5].

Tyrosine kinase inhibitors (TKIs), like Cabozantinib, are associated with a spectrum of dermatologic adverse events that has been described as a “class effect.” These side effects include hand–foot skin reaction (HFSR), alopecia, skin discoloration, pruritus, xerosis, rashes (commonly acneiform, located on the head, neck, chest, and back), scrotal lesions, and stomatitis or mucositis [6,7,8,9]. An analysis of the spontaneous adverse event (AE) reports showed that cutaneous AEs ranked as the second-most commonly reported AEs in patients receiving VEGFR TKIs [10].

## 2. Materials and Methods

This review is based on a systematic literature search of the PubMed (MEDLINE), Google Scholar, Uptodate, and Science Direct databases using the following terms: “Cabozantinib skin toxicity”, “Cabozantinib toxicity”, “Cabozantinib cutaneous toxicity”, “Cabozantinib dermatologic toxicity”. A range of terms associated with skin-related, dermatological, and cutaneous toxicities were used, including specific phrases such as hand–foot syndrome, stomatitis, rash, and several others. Searches were limited to a date range of 2019–2024. Only the ones that had abstracts and were written in English were analyzed in regard to adverse skin-related events associated with Cabozantinib, irrespective of their indications. We included publications reporting clinical trials and safety/toxicity data that were relevant for this review.

The most frequently observed dermatologic toxicities associated with multitargeted kinase inhibitors and their respective locations [11] are summarized below.

In addition, to illustrate these adverse effects, we used images obtained with the informed consent of patients treated with Cabozantinib from the medical practice of the authors, the study being approved by the Ethical Committee of the Clinical Emergency County Hospital of Craiova, Romania (6344/8 February 2024).

## 3. Results and Discussions

### 3.1. Hand–Foot Skin Reaction

Hand–foot skin reaction (HFSR), also named hand–foot syndrome or palmar–plantar erythrodysesthesia (PPE), is a commonly reported sequela of several chemotherapeutic agents and multikinase inhibitors. Factors favoring the development of HFRS due to TKI treatments include female gender, an ECOG (Eastern Cooperative Oncology Group) status of 2 or lower, two or more organs involved, pulmonary or hepatic metastases at baseline, a reference point for white blood cell count exceeding 5.5 × 10⁹ cells/L, and the total time of TKI therapy in cases of HCC [12]. Also, some associated conditions such as diabetes mellitus, fungal infections, and peripheral neuropathy can favor HFSR. Frequency is also higher in Asian populations [13].

**Figure 1:**
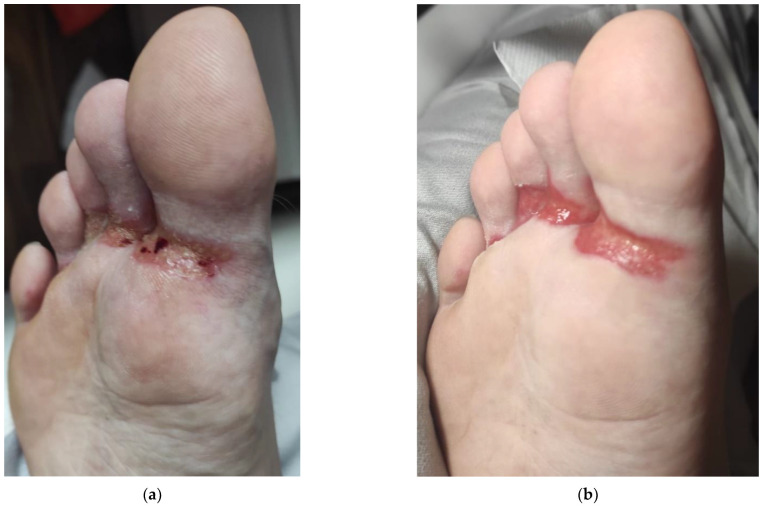
Foot reaction (**a**) before and (**b**) after dermatological treatment.

HFRS is a dermatological condition that sometimes can be painful (Figure 1). The incidence depends on if it is the same drug (Cabozantinib) involved and the tumor type being treated. The calculated incidence of all-grade HFSR, according to a random effects model, was 56.1% for HCC, 50.0% for metastatic medullary thyroid cancer, 42.2% for breast cancer, 30.4% for prostate cancer and glioblastoma, 24.7% for melanoma, and 21.7% for non-small cell lung cancer (*p* < 0.001) [14].

HFS has been identified as a significant adverse effect linked to Cabozantinib treatment. Within the METEOR study, 46% of participants in the Cabozantinib arm experienced HFS, with severe cases (grade 3 or higher) observed in 8% of these patients. Likewise, findings from the CABOSUN trial revealed an HFS occurrence rate of 42%, with grade ≥ 3 cases also affecting 8% of participants. A meta-analysis involving 831 individuals treated with Cabozantinib estimated an overall HFS incidence of around 35%, including a 10% rate for grade 3 cases. As a dose-limiting skin toxicity, HFS may necessitate reducing or pausing the dose, particularly when severe adverse events (grade 3 or above) arise [14,15,16]. Even though HFRS does not directly interfere with overall survival, a limitation of the antitumor effect can result from dose modification or an interruption of the medication.

Another study [17] that used Cabozantinib in Asian patients with metastatic renal cell carcinoma ame to the same results regarding the frequency of adverse events, ranking HFS first (37.5%), followed by anemia.

A meta-analysis regarding the incidence of HFS showed an overall incidence of 35.3%, regardless of the severity of the reaction [18]. Another study, which took place in Italy, included 96 patients with liver cancer, and dermatological AEs (HFSR and skin rash) occurred within 30 days, of when Cabozantinib was started in all but 2 patients [18].

In a phase 3 advanced hepatocellular carcinoma study, HFSR occurred in 46% of the patients treated with Cabozantinib [19].

The exact mechanisms underlying the development of HFSR remain unclear. Proposed explanations include disruptions to pericyte-mediated endothelial survival pathways, which produce alterations in the capillary endothelium at the palmar and plantar level. Other contributing factors may involve the rapid cell turnover in the skin of the palms and soles, combined with temperature differences in distal extremities and the accumulation of toxic drug concentrations in acral eccrine glands. Capillary microtrauma might also allow the leakage of drugs into surrounding tissues, resulting in the build-up of harmful breakdown products [20]. Skin toxicity could stem from one of three main mechanisms: immunologic responses, direct cytotoxic effects, or idiosyncratic reactions. Additionally, some dermatologic reactions may be influenced by the patient’s underlying conditions or drug interactions [21]. Zinc homeostasis, regulated by zinc transporters (Zrt- and Irt-like proteins and Zn transporters) and metallothioneins, appears to play a role in skin differentiation. Case reports and series suggest that zinc deficiency might contribute to the onset of HFSR, while zinc supplementation could potentially alleviate its symptoms [22].

While the incidence of HFRS varies (days to weeks), the symptomatology of this condition is common among TKIs [6]. Usually, the onset of this side effect is between the first 2 to 4 weeks of therapy with TKIs. Chemotherapy- and MKI-induced HFSR each have unique presentations. Chemotherapy-associated HFSR typically presents with diffuse palmoplantar erythema. In contrast, MKI-associated HFSR presents with bilateral, painful, callus-like hyperkeratosis, with some surrounding erythema, most typically localized to areas of increased friction, pressure, and mechanical stress, such as palmoplantar. Rarely, the dorsal aspects of the hands can be affected. Lesions are more visible on the pressure points of both palmar and plantar areas but can also be located on the lateral parts of the extremities of the limbs, the interdigital zones, and the area surrounding the nail, if exposed to mechanical stress. In severe cases, large blisters, widespread desquamation, paronychial inflammation, and ulceration can be seen. The accompanying symptoms consist of redness, edema, pain, and tingling sensation in the affected areas. Other indications may include heightened sensitivity or intolerance to hot or warm objects or fluids, blistering, and dry skin. According to the National Cancer Institute, the clinical manifestations are categorized into three grades. Grade 1 involves minimal skin changes and dermatitis without pain. Grade 2 features more pronounced skin changes, such as peeling, blistering, bleeding, swelling, or hyperkeratosis, along with pain that restricts daily activities. Grade 3 is marked by severe pain, significant skin changes such as blistering or desquamation, and an inability to perform basic self-care activities [23].

Histologically, lesions of MKI-associated HFSR present with epidermal acanthosis and papillomatosis, hyperkeratosis, parakeratosis, dyskeratosis, and the vacuolar degeneration of keratinocytes (subepidermal blisters). Intracytoplasmic eosinophilic bodies and intraepidermal vesicles may be present [24]. Biopsies may reveal superficial telangiectasias, mild superficial perivascular lymphocytic infiltrate, and dysmorphic eccrine cells with cystic changes in the eccrine glands. It is believed that the depth of keratinocyte alteration in the epidermis correlates with the time of exposure to the MKI. Patients on therapy for <30 days demonstrate dyskeratosis in the stratum spinosum and granulosum, whereas patients treated for ≥30 days show hyperkeratosis and parakeratosis in the stratum corneum [25].

HFSR can significantly affect the physical, psychological, and social well-being of patients undergoing treatment with these medications. Although not life-threatening, it may necessitate dose reductions or even the discontinuation of therapy, potentially compromising the treatment’s life-extending benefits. As a result, effective strategies for preventing and managing HFSR are crucial to ensure the consistent use of these drugs and to enhance the health-related quality of life (HRQoL) of affected patients [26]. The primary aim of all management approaches is to preserve or restore patient comfort and HRQoL while allowing the continuation of potentially life-saving therapy for as long as possible.

Educating patients plays a vital role in the early detection and management of HFSR, with simple prophylactic measures as those summarized in Table 1 [11,24,25,26,27] helping to mitigate its severity. Comprehensive guidelines on managing HFS in individuals receiving VEGF pathway inhibitors have been previously reviewed [28,29].

Management options for HFSR are limited, as shown in Table 2 [9,30] but adjusting the dose or discontinuing Cabozantinib usually results in a quick improvement of the lesions. However, this comes with the potential drawback of reducing the effectiveness of cancer treatment, so it should be carefully considered. A proper assessment of the toxicity grade in a clinical setting is essential for effective management. Adverse effect management, according to the severity, as suggested in the literature [11], is listed in Table 3.

For multikinase inhibitors (MKIs), the total dose appears to be associated with both the occurrence and severity of hand–foot skin reaction (HFSR). While a therapeutic response to other targeted agents, such as epidermal growth factor receptor inhibitors, is linked to the development of dermatologic toxicity, there is no evidence to suggest a similar correlation for MKIs (J.P.D., unpublished data, May 2008—as mentioned in [11]).

### 3.2. Stomatitis

Oral toxicity is a common adverse event (AE) associated with multitargeted TKI therapy, affecting 20–35% of patients [31]. In clinical trials, this toxicity is often described as “stomatitis” or “mucositis.” Stomatitis was reported in 22% of patients treated with Cabozantinib in the METEOR trial [15], while dysgeusia, affecting 24% of participants, frequently co-occurred with stomatitis. In the CABOSUN trial, the incidence of oral mucositis and dysgeusia was higher, at 36% and 41%, respectively [16]. Although these AEs rarely require dose reductions or the temporary discontinuation of Cabozantinib, they can significantly diminish patients’ quality of life.

The precise mechanism behind drug-induced stomatitis remains unclear, but it may involve reduced capillary function in the tongue due to VEGFR inhibition or alterations in the oral and gut microbiome. Stomatitis or oral mucositis may present as oral sensitivity, dysphagia (difficulty swallowing), dry mouth, taste changes, mucosal tenderness, mild erythema, and painful inflammation. In some cases, ulcers can develop as discrete linear lesions in nonkeratinized mucosal areas, such as the labial and buccal mucosa, tongue, floor of the mouth, and soft palate [32]. Oral AEs typically emerge within the first few months of treatment [19].

Oral dysesthesia, another related condition, represents sensory perceptions in the oral cavity, e.g., a burning feeling, despite no visible abnormalities in the oral mucosa [33]. Patients with oral dysesthesia may report increased sensitivity or discomfort even in the absence of dysgeusia or dry mouth. The term “oral dysesthesia” has been suggested for better accuracy in describing this condition [34], which is commonly observed with VEGFR-directed multi-target TKIs like Cabozantinib. Symptoms typically arise within 0.5–1.4 months after starting treatment [35]. Recommended preventive measures for these AEs are detailed in Table 4.

Adverse events of stomatitis based on the severity are described in Table 5 according to the National Cancer Institute Common Terminology Criteria for Adverse Events (NCl-CTCAE) [24]. Methods for the management of oral toxicity are listed in Table 6 [9].

Grade 1 toxicities are characterized by either no symptoms or mild symptoms. Grade 2 toxicities involve moderate pain or ulcers that do not affect the ability to eat. Grade 3 toxicities are marked by severe pain that disrupts oral intake. Typically, grade 3 stomatitis requires dose interruption and often dose reduction, while grade 1–2 toxicities can usually be managed without adjusting the dosage.

### 3.3. Rash

**Figure 2:**
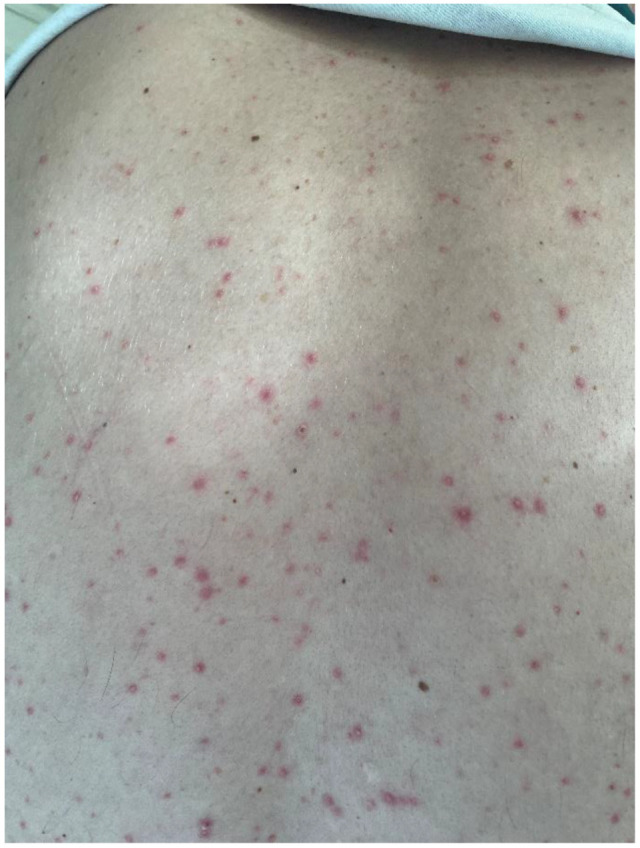
Papulo-pustulous rash.

Multikinase inhibitors are known to cause dose-dependent rashes, which commonly appear during the initial weeks of therapy but do not indicate treatment efficacy. These side effects typically present with varied forms [36], such as macules, papules, or maculopapules, which may be red and/or itchy. Rashes are most often located on the torso, limbs, or genital area (Figure 2).

The management of rashes occurring during TKI therapy depends on the toxicity grade.

Grade 1 toxicity involves macules or papules covering less than 10% of the body surface area (BSA), with or without symptoms such as itching, burning, or tightness.

Grade 2 toxicity is defined by 10–30% of the BSA affected by macules or papules, with or without symptoms (e.g., itching, burning, tightness), which may limit instrumental activities of daily living (ADL), or by rashes covering more than 30% of the BSA with mild symptoms.

Grade 3 toxicity involves macules or papules affecting over 30% of the BSA, along with moderate to severe symptoms that impair self-care ADL.

Patients experiencing rashes are advised to use fragrance-free soaps, apply moisturizers, and wear loose, comfortable clothing. Mild cases may benefit from topical corticosteroids and antihistamines.

If the rash is severe, dose adjustments or a temporary break from TKI therapy should be considered. Most rashes resolve within six weeks and may even improve spontaneously, as observed in some cases of facial or scalp erythema.

### 3.4. Hair Changes

MKI-related hair changes impact texture, density, and color, resulting in brittle hair with kinking, depigmentation, and slower growth. These changes are generally reversible after discontinuing the drug [37]. Hair and/or widespread skin depigmentation occurred in 44% of patients, with a median onset of 11.4 weeks following the start of Cabozantinib. Hair discoloration is commonly observed on the scalp, eyelashes, and eyebrows [3]. Patients on prolonged Cabozantinib therapy may have an increased risk of heightened photosensitivity, including sunburn on areas exposed to the sun. Hair and skin depigmentation typically reverses within weeks of stopping treatment. Certain processes, such as the development, migration, and survival of melanocytes, are regulated by c-KIT, a protein inhibited by Cabozantinib [3]. Patients are advised to avoid extended exposure to UVA and UVB rays, use broad-spectrum sunscreen, and wear UV-protective clothing, hats, and sunglasses. If needed, symptomatic treatment with topical or systemic corticosteroids may be beneficial [38].

### 3.5. Xerosis

Xerosis appears in approximately 20% of the cases and primarily develops on distal extremities. Interestingly, all patients with xerosis also developed HFSR. Acral involvement resulted in painful fissures and affected the functionality of the area. The frequent use of 5% to 10% urea-moisturizers usually resolves the xerosis [3].

### 3.6. Scrotal Erythema

**Figure 3:**
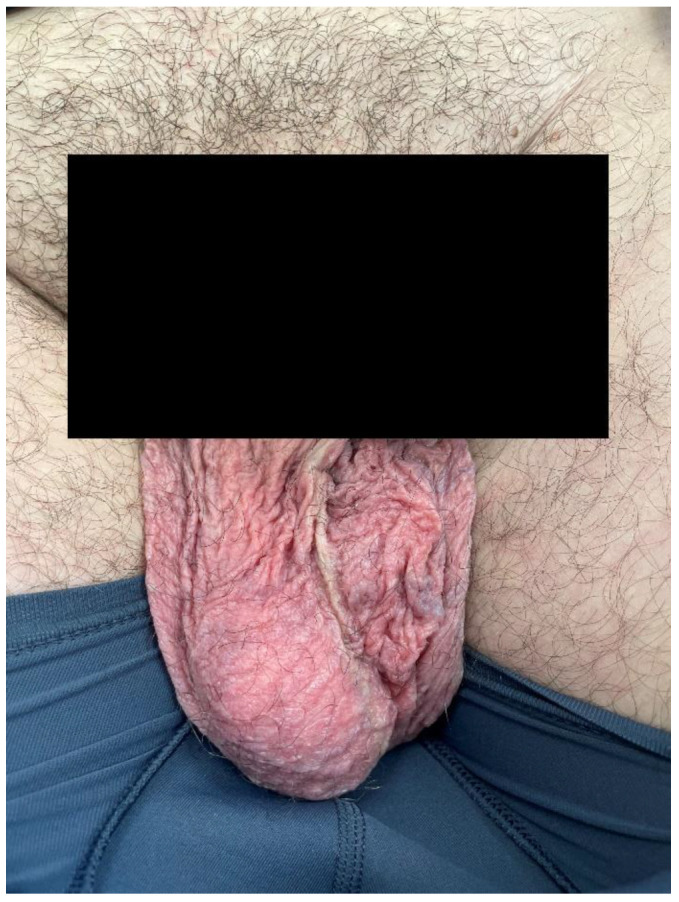
Scrotal erythema.

Scrotal erythema (Figure 3) is seen in approximately one quarter of the male patients, usually after 5 weeks of treatment. The areas affected are those predisposed to friction and trauma such as the inguinal area and scrotal skin. The inhibition of VEGF and the hypoxia-inducible factor 1-α may contribute to this adverse effect [39]. Clinically, it presents as erythematous macules. Management involves using athletic supporters to minimize friction and applying barrier ointments or pastes such as zinc oxide and menthol. Additionally, topical steroids can be utilized [3]. A single case report describes scrotal ulcerations induced by Cabozantinib in a patient with kidney cancer [40].

### 3.7. Subungual Splinter Hemorrhages

Subungual splinter hemorrhages were identified in 12% of cases [3], presenting as one or more asymptomatic, infracentimetric longitudinal brown to black lines beneath the distal nail plate. This phenomenon is linked to the antiangiogenic effects of TKIs, as the VEGFR blockade may inhibit the repair of nail-bed capillaries, which are prone to frequent microinjuries at the fingertips [41].

### 3.8. Impaired Healing Due to Antiangiogenic Drug Use

Impaired wound healing is a potential complication following treatment with Cabozantinib [30]. The inhibition of angiogenesis disrupts normal skin homeostasis, and drugs targeting VEGF or VEGFR can delay the wound healing process. One study reported wound dehiscence in 2.8% of patients treated with Cabozantinib [42]. It is recommended to discontinue these drugs 24–48 h prior to surgery and wait 3–4 weeks post-surgery before resuming treatment [43]. Ideally, therapy should stop until the healing process is complete.

Despite the dermatological side effects associated with Cabozantinib, a study on patients with renal cell carcinoma investigated the link between skin toxicity and clinical efficacy. The findings indicated that cutaneous adverse events were associated with improved overall survival and treatment response in patients with mRCC receiving immunotherapy and/or TKIs [44]. Additionally, conditions such as hypertension and mucositis/HFSR may correlate with better outcomes. Further research is needed to validate these observations [45].

## 4. Conclusions

Multikinase inhibitors, such as Cabozantinib, have become standard treatments for medullary thyroid cancer, hepatocellular carcinoma, and advanced renal cell carcinoma. Skin reactions are among the most common adverse effects linked to Cabozantinib. These AEs notably impact patients’ quality of life and drug adherence. The effective management of dermatologic adverse events necessitates a multidisciplinary collaboration between dermatology and oncology teams. It is essential for dermatologists to identify these cutaneous reactions associated with Cabozantinib, as they pose a significant therapeutic challenge in optimizing the efficacy of targeted cancer therapy.

The findings of this review showcase the need for further research on factors influencing HFSR in patients treated with Cabozantinib and the importance of exploring molecular mechanisms, evaluating management protocols, and conducting pharmacovigilance studies to improve long-term safety and treatment efficacy, all while promoting better patient education for increased therapeutic adherence.

## Figures and Tables

**Table 1:** Prophylaxis of hand and foot skin reaction.

– Eliminate existing hyperkeratotic areas or calluses. – Apply emollients with 10% urea, use topical exfoliants, and protect areas prone to pressure. – Refrain from using hot water. – Minimize friction and pressure on the hands and feet. – Limit exposure to direct sunlight and avoid unprotected exposure to cold. – Avoid scented, foaming cleansers and alcohol-based hand sanitizers. – Opt for thick cotton socks, gloves, cushioned insoles, and properly fitting footwear.

**Table 2:** Management of hand and foot skin reaction.

Preventative measures should be continued Use emollients containing 20–40% urea Use salicylic acid, ammonium lactate or alpha hydroxyl acid, tazarotene 0.1% cream, and fluorouracil 5% cream to soften and exfoliate hyperkeratotic and callused areas Use a solution of recombinant human basic endothelial or fibroblast growth factor or vitamin B on skin areas showing minor wear Use magnesium sulphate cooling baths for hands and feet to reduce pain and soften calluses Use topical treatments such as bethametosone, clobetasol 0.05%, and topical lidocaine to relief symptoms and treat erythematous/inflammatory lesions Use oral analgesia such as nonsteroidal anti-inflammatory drugs (NSAIDS), pregabalin, and opioids if topical treatment is ineffective Use equal parts vinegar and water for 10 min each day on cracked skin to prevent infection Refer to dermatology or podiatry for significant dermatological symptoms

**Table 3:** Management of hand and foot syndrome according to the severity grade.

Grade 1	Refrain from using hot water. Apply moisturizing creams regularly. Use keratolytic treatments like urea 20–40%, salicylic acid 6%, tazarotene 0.1%, or fluorouracil 5% creams on hyperkeratotic areas twice daily. Wear cotton gloves and socks at night. No dose adjustments are advised. A follow-up visit to the clinic is recommended after two weeks.
Grade 2	The prior treatment should be maintained along with the following: Apply clobetasol 0.05% ointment to affected erythematous areas twice daily. Use topical analgesics like 2% lidocaine and systemic pain relievers (e.g., nonsteroidal anti-inflammatory drugs, codeine, pregabalin). Consider reducing the dose to 50% of the full dose for at least 7 days, up to 28 days, until the HFSR improves to grade 1 or 0, then return to the full dose.
Grade 3	Maintain the previous measures along with the following: Discontinue treatment for a minimum of 7 days until the HFSR improves to grade 1 or 0, then resume treatment at 50% of the full dose. Monitor the patient for any signs of toxicity. If toxicity does not return, dose escalation may be considered until the full dose is restored.

**Table 4:** Prophylaxis of oral toxicity due to Cabozantinib.

Regular dental examination, along with replacement or removal of ill-fitting dentures. Avoid alcohol, tobacco and salty, spicy or acid food. Maintain good oral hygiene by brushing teeth with a soft-bristled toothbrush and fluoride toothpaste after eating and before bedtime. Floss daily and use alcohol-free mouthwash four times per day. Stay hydrated to keep the mouth moist.

**Table 5:** The NCI-CTCAE grading system [24].

Grade 1	Asymptomatic or mild symptoms; intervention not indicated
Grade 2	Moderate pain not interfering with oral intake; modified diet indicated
Grade 3	Severe pain; interfering with oral intake
Grade 4	Life-threatening consequences; urgent intervention indicated
Grade 5	Death

**Table 6:** Management of the oral toxicity according to the severity grade.

Grade 1 or 2	Medicated mouthwash to treat oral mucositis, which may include nystatin, diphenhydramine, viscous lidocaine, dyclonine, aluminum hydroxide, or magnesium hydroxide, and occasionally corticosteroids, or mouth rinses with 0.9% saline or 0.9% sodium bicarbonate. Sucralfate mouthwash and olive oil mouthwash. Mucosal coating agents, topical anesthetics, and/or benzydamine HCl. Low-level laser therapy and intralesional steroid injections. Triamcinolone acetonide ointment, doxycycline mouthwash (two tablets dissolved in 250 mL water), and erythromycin (250–350 mg). No dose modification is required.
Grade 3	All the above plus erythromycin 500 mg or minocycline 100 mg and systemic corticosteroid 30–60 mg pulse therapy or 1 mg/kgc 1 week with tapering. Dose interruption until recovery to <grade 2; first episode: try to restart at full dose; second episode: reduce the dose until the AE has reduced in severity by 1 grade.
Grade 3–4	Continue the treatment described above. As there is a risk of death, consider a permanent reduction or interruption.

## Data Availability

The data presented in this study are available on request from the corresponding author.
